# ^99m^Tc-DPD scintigraphy in immunoglobulin light chain (AL) cardiac amyloidosis

**DOI:** 10.1093/ehjci/jeab095

**Published:** 2021-07-13

**Authors:** Candida Cristina Quarta, Jiexin Zheng, David Hutt, Simona F Grigore, Richa Manwani, Sajitha Sachchithanantham, Shameem A Mahmood, Carol J Whelan, Marianna Fontana, Ana Martinez-Naharro, Liza Chacko, Helen J Lachmann, Julian D Gillmore, Claudio Rapezzi, Philip N Hawkins, Ashutosh D Wechalekar

**Affiliations:** 1 National Amyloidosis Centre, University College London (Royal Free Campus), Rowland Hill Street, London NW3 2PF, UK; 2 Alexion Pharmaceutical LTD, Stockley Park, 3 Furzeground Way, Hayes, Uxbridge, London UB11 1EZ, UK; 3 Cardiovascular Center, University Hospital of Ferrara, Ferrara, Italy; 4 Maria Cecilia Hospital, GVM Care & Research, Cotignola, RA, Italy

**Keywords:** amyloidosis, light chain, cardiomyopathy, 99mTc-DPD scintigraphy, diagnosis, prognosis

## Abstract

**Aims:**

Technetium-99m-labelled 3,3-diphosphono-1,2-propanodicarboxylic acid (^99m^Tc-DPD scintigraphy) is recognized as highly accurate for the non-invasive diagnosis of transthyretin (ATTR) cardiac amyloidosis (CA). A proportion of patients with immunoglobulin light chain (AL) CA have also been reported to show cardiac ^99m^Tc-DPD uptake. Herein, we assessed the frequency and degree of cardiac ^99m^Tc-DPD uptake and its clinical significance among patients with AL CA.

**Methods and results:**

Between 2010 and 2017, 292 consecutive patients with AL CA underwent ^99m^Tc-DPD scintigraphy and were included in this study: 114 (39%) had cardiac ^99m^Tc-DPD uptake: grade 1 in 75%, grade 2 in 17%, and grade 3 in 8% of cases. Patients with cardiac ^99m^Tc-DPD uptake had poorer cardiac systolic function and higher N-terminal pro-brain natriuretic peptide. No differences were noted in cardiac magnetic resonance parameters between patients with and without cardiac ^99m^Tc-DPD uptake (*N* = 19 and 42, respectively). Patients with cardiac ^99m^Tc-DPD uptake showed a trend to worse survival than those with no uptake (log-rank *P* = 0.056). Among 22 patients who underwent serial ^99m^Tc-DPD scintigraphy, 5 (23%) showed reduction in the grade of cardiac uptake.

**Conclusions:**

In this large cohort of patients with AL CA, ^99m^Tc-DPD scintigraphy ∼40% of cases showed cardiac uptake, including grade 2–3 in 10% of all patients (25% of those with cardiac ^99m^Tc-DPD uptake). Cardiac ^99m^Tc-DPD uptake was associated with poorer cardiac function and outcomes. These data highlight the critical importance of ruling out AL amyloidosis in all patients with cardiac ^99m^Tc-DPD uptake to ensure such patients are not assumed to have ATTR CA.

## Introduction

Systemic amyloidoses are rare disorders characterized by extracellular accumulation of fibrillary proteins leading to loss of normal tissue architecture and organ function.^1^ Immunoglobulin light chain (AL) amyloidosis is the most frequent type of systemic amyloidosis. The second most common type is the wild-type form of transthyretin amyloidosis (ATTR) which is being recognized increasingly.^2^ Differential diagnosis is crucial given the different outcomes and treatments.^3^

AL amyloidosis is caused by a clone of plasma cells producing abnormal and unstable immunoglobulin lights chains resulting in formation of AL amyloid fibril deposits.^1^ Although amyloid fibrils can deposit in multiple organs, cardiac involvement occurs in 50–70% of patients with AL amyloidosis and is the main determinant of prognosis.^3^ Indeed, the most widely accepted prognostic stratifications in AL amyloidosis (the original staging system reported by the Mayo group as well as its subsequent modifications) are based on the extent of cardiac involvement determined by the serum cardiac biomarkers N-terminal pro-brain natriuretic peptide (NT-proBNP) and cardiac troponin T; patients with a very high NT-proBNP and high cardiac troponin-T (Mayo stage 3b) have a median survival of 5 months.^4–6^

In recent years, the myocardial uptake of the radiotracer technetium-99m-labelled 3,3-diphosphono-1,2-propanodicarboxylic acid (^99m^Tc-DPD) or similar bone tracers such as pyrophosphate (PYP) have been validated and increasingly used in the non-biopsy diagnostic pathway of ATTR amyloid cardiomyopathy. Patients with ATTR cardiac amyloidosis (CA) almost always show a moderate-to-strong ^99m^Tc-DPD cardiac uptake (visual grade 2–3, representing greater uptake in the heart than in bones), while patients with non-amyloid cardiomyopathies show no evidence of ^99m^Tc-DPD cardiac uptake (visual score 0).^7–10^

There unfortunately remains a wide perception that patients with AL amyloidosis do not demonstrate significant cardiac uptake on these scans despite several previous small studies showing the contrary, typically with mild (grade 1) myocardial localization.^7^^,^^10^ However, the role and utility of bone scintigraphy tracers (^99m^Tc-DPD/PYP) in AL CA have not been systematically studied nor fully established.

In a large cohort of consecutive patients with AL CA, we aimed to assess the presence and degree of cardiac ^99m^Tc-DPD uptake and investigated its clinical significance. We also aimed at investigating the possible mechanisms underlying the presence of cardiac ^99m^Tc-DPD uptake in the different CA aetiologies.

## Methods

### Setting and study design

We conducted a retrospective analysis of all consecutive patients diagnosed with AL CA at the National Amyloidosis Centre between June 2010 and November 2017, who also underwent ^99m^Tc-DPD scintigraphy as part of a clinical suspicion of CA in order to distinguish non-invasively between AL, ATTR, or other forms of systemic amyloidosis with cardiac involvement. Only patients with a confirmed diagnosis of AL CA were included in this study. Patients with coexistent ATTR and AL cardiac amyloid deposits were excluded from the analysis.

All patients underwent a comprehensive evaluation protocol comprising of 12-lead electrocardiogram (ECG), echocardiogram, cardiac magnetic resonance (CMR), biochemical tests including serum and urine immunofixation, electrophoresis and serum-free light chain assay, ^123^I-serum amyloid P component scintigraphy,^11^^99m^Tc-DPD scintigraphy, TTR gene sequencing,^12^ and where necessary, sequencing of other hereditary amyloidosis genes. 

For each patient the presence and degree of ^99m^Tc-DPD cardiac uptake was defined.^9^ Patients were stratified into two groups according to the absence (i.e. visual score 0) or presence (visual score ≥1) of cardiac uptake by ^99m^Tc-DPD scintigraphy and were compared in terms of clinical/instrumental profiles at baseline (i.e. first evaluation at our centre) and clinical outcome.

Since chemotherapy can affect the natural history of AL amyloidosis,^13^ we only included newly diagnosed patients who were treatment-naïve at the time when ^99m^Tc-DPD scintigraphy and other assessments were performed.

In order to evaluate the clinical significance of cardiac ^99m^Tc-DPD uptake, we investigated the presence of correlations between cardiac ^99m^Tc-DPD uptake and other imaging and laboratory measures of interest as well as patients’ outcome.

All patients were managed in accordance with the Declaration of Helsinki and provided a generic informed consent for anonymous publication of scientific data. Of note, 188 of the 292 patients included in the present study were also included in the previous multicentre study addressing the sensitivity and specificity of ^99m^Tc-DPD scintigraphy to identify ATTR cardiomyopathy.^9^

### Diagnostic definitions

Diagnostic definitions for amyloidosis, AL aetiology, and cardiac involvement can be found in the Supplementary data online.^9^^,^^14–17^

### Cardiac investigations

A list and description of the cardiac investigation utilized, including ECG, echocardiography, and CMR can be found in the Supplementary data online.^18–21^

### ^99m^Tc-DPD scintigraphy

Patients were administered 700 MBq of ^99m^Tc-DPD intravenously and imaged 3 h later on a General Electric (GE) Infinia Hawkeye 4 or GE Discovery 670 hybrid gamma camera. Whole body images were acquired at a scan speed of 10 cm/min using low energy high-resolution collimators and were immediately followed by a SPECT-CT (single-photon emission computed tomography with a low-dose, non-contrast CT scan) of the heart. The expected radiation dose from the entire procedure was 6.7 mSv per patient.^22^

Intensity of myocardial uptake on the planar 99mTc-DPD scan was categorized as 0–3 according to the grading system described by Perugini *et al.*^23^: grade 0—no cardiac uptake and normal bone uptake; grade 1—mild cardiac uptake, but less intense than the bone signal; grade 2—moderate cardiac uptake with intensity similar or greater than the bone signal; grade 3—strong cardiac uptake with much attenuated or absent bone signal.

### Follow-up

Follow-up visits were planned for every 6 months (or more frequently if clinically appropriate). Follow-up was closed in January 2019; for patients who had not attended a visit in the last 6 months, vital status was ascertained by telephone and/or by contacting referring physicians. Overall survival was calculated from diagnosis to last follow-up or death.

### Statistical analysis

Summary statistics were expressed as mean ± standard deviation, median (interquartile range), or numbers (percentages). We used the unpaired *t*-test or the Pearson *χ*^2^ test for comparisons of baseline data between patients with and without ^99m^Tc-DPD cardiac uptake.

We tested a priori selected variables chosen for their potential clinical relevance and/or significant differences between patients with or without cardiac ^99m^Tc-DPD uptake in multivariate logistic regression models constructed to assess their association with the presence of cardiac ^99m^Tc-DPD uptake. Skewed independent variables were log-transformed. Multivariate logistic regression models were reported as odds ratio (OR) and 95% confidence interval (CI).

Overall survival was analysed with Kaplan and Meier curves. To explore risk factors that could be associated with all-cause mortality, univariate Cox regression analysis was initially performed using clinical, laboratory, and instrumental variables. Multivariable analysis was then performed by entering the model a set of variables that were considered significant on univariate analysis (*P* < 0.1) or on the basis of their potential clinical or pathophysiological relevance. In the presence of highly correlated parameters, only one variable was included in the final model.

Analyses were conducted using STATA version 15.1 (StataCorp, College Station, TX, USA). Values of *P*-value <0.05 were considered significant.

## Results

Of 926 patients consecutively diagnosed with AL CA at our centre between 2010 and 2017, a total of 292 consecutive patients also underwent ^99m^Tc-DPD scintigraphy and were included in this study. A total of 178 (61%) patients had no cardiac uptake by ^99m^Tc-DPD scintigraphy while 114 (39%) showed cardiac uptake. Of the 114 with ^99m^Tc-DPD cardiac uptake, 85 (75%) had a visual score = 1; 20 (17%) had visual score = 2, and 9 (8%) had a visual score = 3.

*Table 1* summarizes the main baseline characteristics in the study population according to the presence or absence of ^99m^Tc-DPD cardiac uptake. In both groups, <1/3 of patients had isolated cardiac involvement. There was no significant difference between the two groups in terms of the type of the involved serum-free light chain as well as of the difference between the involved and uninvolved light chain.

**Table 1:** Main baseline characteristics of the study population according to the presence or absence of ^99m^Tc-DPD cardiac uptake

Baseline characteristics	No cardiac ^99m^Tc-DPD uptake (*n* = 178)	Cardiac ^99m^Tc-DPD uptake (*n* = 114)	*P*-value
Male gender, *n* (%)	134 (75)	86 (75)	0.98
Age at diagnosis (years)	69 ± 9	67 ± 9	0.07
Supine systolic blood pressure (mmHg)	116 ± 19	114 ± 18	0.38
Disease duration (months)	12 (6–18)	9 (6–18)	0.36
NYHA class, *n*/*N* (%)			0.76
I	18/170 (10)	9/112 (8)	
II	104/170 (58)	69/112 (61)	
III	42/170 (24)	27/112 (24)	
IV	6/170 (3)	7/112 (6)	
Lambda light chain plasma cell dyscrasia, *n* (%)	140 (79)	85 (75)	0.42
Creatinine (mMol/L)	103 (81–127)	106 (84–148)	0.21
eGFR (mL/min/m^2^)	62 (47–83)	59 (39–74)	0.1
Proteinuria (g/24h)	0.6 (0.14–2.35)	0.4 (0.16–1.5)	0.52
Serum albumin (g/L)	37.7 ± 6.8	39.0 ± 6.2	0.1
Total serum bilirubin (μmol/L)	10 (7–15)	12 (9–17)	**0.001**
NT-proBNP (ng/L)	4729 (2243–9949)	8191 (3856–15 839)	**<0.001**
NT-proBNP >8500 ng/L, *n* (%)	54 (31)	53 (46)	**0.006**
Cardiac troponin T (ng/mL)	85.5 (48–137)	104 (67–221)	**0.002**
Mayo stage 3, *n* (%)	124 (70)	94 (83)	**0.014**
Extra-cardiac organ involvement, *n* (%)			
Kidney	85 (48)	52 (46)	0.72
Liver	20 (11)	12 (11)	0.87
Autonomic neuropathy	25 (14)	19 (17)	0.52
Peripheral neuropathy	21 (12)	20 (18)	0.16
Gastro-intestinal	17 (10)	14 (12)	0.44
Soft tissues	66 (37)	34 (30)	0.22
Lymph nodes	2 (1)	NA	0.26
Lungs	1 (1)	2 (2)	0.32
Number of organs affected, *n* (%)			0.61
1	36 (20)	29 (25)	
2	75 (42)	39 (34)	
3	46 (26)	30 (26)	
4	15 (8)	12 (11)	
5	5 (3)	3 (3)	
6	1 (1)	0 (0)	
7	0 (0)	1 (1)	
Low QRS voltage, *n*/*N* (%)	81/174 (47)	59/111 (53)	0.28
Sinus rhythm, *n*/*N* (%)	138/172 (77)	93/108 (82)	0.21
LV septum thickness (mm)	15 ± 2	15 ± 2	0.65
LV posterior wall thickness (mm)	15 ± 2	15 ± 2	0.95
LV ejection fraction (%)	52 ± 10	48 ± 11	**0.002**
E/E′ ratio	19 ± 9	20 ± 9	0.39
S′ wave velocity (lateral wall) (cm/s)	0.06 (0.05–0.07)	0.05 (0.04–0.07)	**0.04**
LV global longitudinal strain (%)	−12 ± 4	−10 ± 4	**0.001**
ECV by CMR (%)	51 ± 10 (*n* = 42)	51 ± 8 (*n* = 19)	0.83
Native T1 (ms)	1142 (1106–1161) (*n* = 46)	1156 (1106–1176) (*n* = 21)	0.36
Transmural pattern of LGE, *n*/*N* (%)	26/45 (58)	11/19 (58%)	0.65
Amyloid deposition on ^123i^SAP scintigraphy, *n* (%)			
Liver	30 (17)	14 (12)	0.29
Spleen	75 (42)	45 (44)	0.65
Kidney	33 (19)	22 (19)	0.87
Bones	12 (7)	12 (11)	0.25

CMR, cardiac magnetic resonance; ECV, extracellular volume; eGFR, estimated glomerular filtration rate; LGE, late gadolinium enhancement; LV, left ventricular; NA, not applicable; NT-proBNP, N-terminal pro-brain natriuretic peptide; NYHA, New York Heart Association; SAP, serum amyloid P component.

At baseline, patients with cardiac ^99m^Tc-DPD uptake had worse systolic function and higher cardiac biomarkers (and a higher proportion of Mayo stage 3) than those with no cardiac tracer uptake (*Table 1*).

Sixty-seven patients underwent CMR at baseline, 46 in the group without and 21 in the group with cardiac ^99m^Tc-DPD uptake, which was a non-contrast study in 6 cases (estimated glomerular filtration rate was <30 mL/min/m^2^). There were no differences between the two groups in extracellular volume (ECV) or native T1; almost 60% of patients in both groups had a transmural pattern of late gadolinium enhancement. Among the 61 patients who underwent CMR with gadolinium, ECV did not correlate with the intensity of cardiac ^99m^Tc-DPD uptake (OR 0.99, CI 0.94–1.05; 4*P* = 0.83).

Of the 114 patients with cardiac ^99m^Tc-DPD uptake, 22 (19%) had at least one repeat ^99m^Tc-DPD scan during their follow-up. After 29 (13.9–43.9) months, 16 (73%) remained stable, 1 (4%) showed worsening, and 5 (23%) showed improvement in the grade of ^99m^Tc-DPD uptake. There were no significant differences in clinical, laboratory, and imaging findings between patients with and without regression of cardiac ^99m^Tc-DPD uptake.

**Figure 1:**
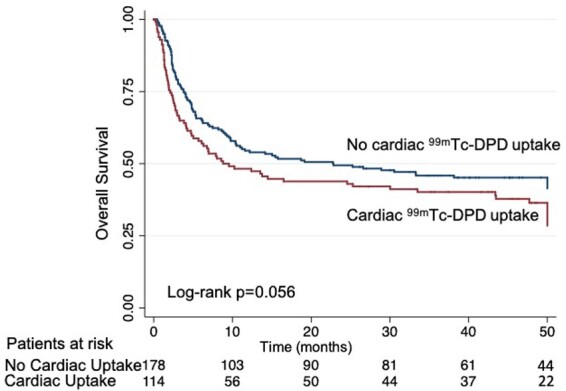
^99m^TcDPD scintigraphy (3 hours’ image acquisition) in a patient with stage 3a light chain (AL) cardiac amyloidosis. While at diagnosis (left) the cardiac and bone uptake were, respectively, intense and attenuated (visual score = 2), after 2 years (right) the degree of cardiac uptake has substantially reduced, while the bone uptake has increased (visual score = 1).

*Figure 1* shows an example of cardiac ^99m^Tc-DPD uptake regression in a patient with AL CA. 

At univariate analysis (*Table 2*), left ventricular (LV) longitudinal strain, LV ejection fraction, serum bilirubin, cardiac troponin, and serum NT-proBNP levels were all associated with cardiac ^99m^Tc-DPD uptake. At multivariable analysis (*Table 2*), only serum bilirubin levels were independently associated with the presence of cardiac ^99m^Tc-DPD uptake. 

**Table 2:** Univariate and multivariate logistic regression analysis for factors associated with the presence of ^99m^Tc-DPD cardiac uptake

Univariate	OR	95% CI	*P*-value
Global LV longitudinal strain (per 1%)	1.10	1.04–1.17	**0.002**
LV ejection fraction (per 1%)	0.96	0.94–0.99	**0.002**
NT-proBNP (per 1 ng/L)	1.43	1.15–1.79	**0.001**
Cardiac troponin T (per 1 ng/mL)	1.64	1.22–2.19	**0.001**
Bilirubin (per 1 mg/dL)	1.99	1.34–2.96	**0.001**
**Multivariate**			
Global LV longitudinal strain (per 1%)	1.06	0.98–1.14	0.11
LV ejection fraction (per 1%)	0.98	0.96–1.01	0.29
NT-proBNP (per 1 ng/L)	1.03	0.72–1.46	0.87
Cardiac troponin T (per 1 ng/mL)	1.27	0.83–1.94	0.26
Bilirubin (per 1 mg/dL)	1.60	1.03–2.47	**0.03**

CI, confidence interval; LV, left ventricular; NT-proBNP, N-terminal pro-brain natriuretic peptide; OR, odds ratio; p values of signifiance in **bold**

### Outcomes

Median (interquartile range) follow-up was 15 (3–49) months [23 (4–54) months in patients with no ^99m^Tc-DPD cardiac uptake and 9 (2–47) months in those with ^99m^Tc-DPD cardiac uptake].

All patients received treatment for the underlying haematological disorder, consisting of velcade- or thalidomide-based regimens, with no differences between patients with and without ^99m^Tc-DPD cardiac uptake.

During the study period, there were 177 (61%) deaths (101 among patients with no ^99m^Tc-DPD cardiac uptake and 76 among patients with ^99m^Tc-DPD cardiac uptake).

**Figure 2:**
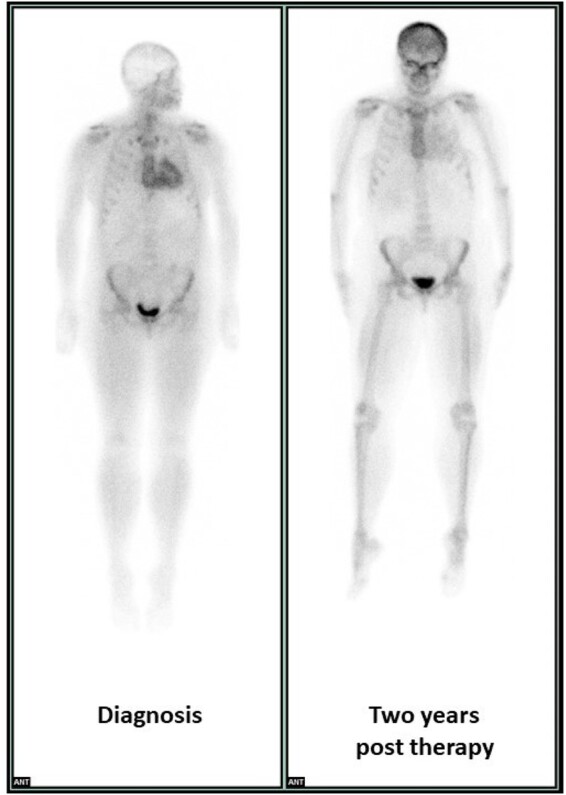
Overall survival in patients with AL cardiac amyloidosis according to the presence or absence of cardiac ^99m^Tc-DPD uptake.

*Figure 2* shows the unadjusted overall survival in patients with vs. those without cardiac ^99m^Tc-DPD uptake. Overall, patients showing cardiac ^99m^Tc-DPD uptake showed a trend to worse survival (borderline statistical significance, log-rank *P* = 0.056). Unadjusted overall survival at 6 months, 1, 2, and 3 years was 66%, 54%, 49%, and 46% for patients with no cardiac ^99m^Tc-DPD uptake; 58%, 48%, 44%, and 40% for those with ^99m^Tc-DPD cardiac uptake.

*Table 3* reports the results of the multivariate analysis for risk factors associated with all-cause mortality. The presence of cardiac ^99m^Tc-DPD uptake was not associated with an increased risk of death, while cardiac biomarkers were unfavourable predictors of all-cause mortality as was the serum bilirubin concentration.

**Table 3:** Multivariable analysis of risk of death resulting from any cause

	HR	95% CI	*P*-value
Age (each incremental year)	1.02	0.99–1.04	0.10
Presence of cardiac ^99m^Tc-DPD uptake	1.11	0.81–1.53	0.52
Global LV longitudinal strain (each incremental 1%)	1.06	0.99–1.10	0.06
NT-proBNP (each incremental ng/L)	1.40	1.12–1.76	**0.004**
Cardiac troponin T (each incremental ng/mL)	1.32	1.01–1.73	**0.043**
Bilirubin (each incremental mmol/L)	1.84	1.40–2.41	**<0.001**

CI, confidence interval; HR, hazard ratio; LV, left ventricular; NT-proBNP, N-terminal pro-brain natriuretic peptide; p values of signifiance in **bold**.

## Discussion

This is the first study to focus specifically on the role of ^99m^Tc-DPD scintigraphy in patients with AL CA. Our study provides two main findings: (i) 40% of patients with AL CA shows evidence of cardiac ^99m^Tc-DPD uptake, with a grade ≥2 (i.e. at least moderate) cardiac uptake in 1/4 of cases and (ii) in AL CA, ^99m^Tc-DPD myocardial uptake is associated with higher cardiac biomarkers, worse cardiac function, and a suggestion of worse overall survival beyond conventional prognostic markers.

Over the past three decades, radionuclide ‘bone’ scintigraphy using technetium labelled bisphosphonates has been increasingly reported to localize to cardiac amyloid deposits.^7–9^^,^^22–24^ Systematic evaluation of bone scintigraphy has shown that ^99m^Tc-DPD, ^99m^Tc-labeled PYP, and ^99m^Tc-labeled-hydroxymethylene diphosphonate (HMDP) are remarkably sensitive and specific for imaging ATTR cardiac amyloid.^9^

In a small study from 2005, Perugini *et al.*^23^ reported evidence of cardiac ^99m^Tc-DPD uptake in patients with ATTR (*n* = 17) but not in those with AL (*n* = 8) CA or in unaffected controls (*n* = 10). In an extended cohort of 79 patients with CA, the same group subsequently reported a grade ≥2 of cardiac ^99m^Tc-DPD uptake in all patients with ATTR CA and a grade 1–2 cardiac ^99m^Tc-DPD uptake in about one-third of patients with AL CA.^7^

In the largest multicentre study so far testing the diagnostic performance of various bone tracers, Gillmore *et al.*^9^ reported a sensitivity of >99% and a specificity of 86% to diagnose ATTR CA, the lower specificity resulting largely from low-grade cardiac uptake in patients with other aetiologies of CA.

**Figure 3:**
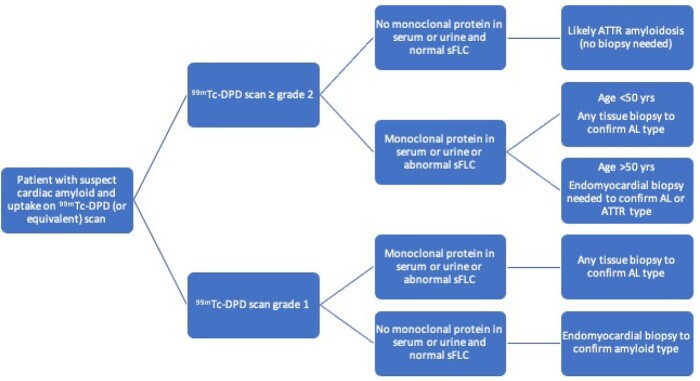
Diagnostic algorithm in patients with suspect cardiac amyloidosis and evidence of cardiac uptake on 99mTc-DPD scintigraphy (or equivalent bone scanning). While with a ^99m^TcDPD grade 2 or 3 the absence of a monoclonal protein in serum/urine is diagnostic of ATTR cardiomyopathy, its presence requires more investigations, and in particular the differential diagnosis between ATTR and AL amyloidosis via endomyocardial biopsy in patients older than 50 years, that are at higher risk of ATTR. A ^99m^TcDPD grade 1 in absence of a monoclonal protein in serum/urine still requires an endomyocardial biopsy to understand the nature of the underlying cardiomyopathy/amyloidosis.

Our study included the largest population of patients with AL CA studied with a single bone tracer. Our finding that 40% of AL patients had cardiac ^99m^Tc-DPD uptake, which was at least moderate in 25% of cases (10% of the overall population analysed), is especially relevant when considering that most studies have focused on the high diagnostic accuracy of cardiac uptake by bone scintigraphy in ATTR amyloidosis. Crucially and concerningly, there is an ever-increasing trend for patients referred as having ATTR CA based on the combination of suggestive cardiac imaging and positive ^99m^Tc-DPD cardiac uptake. This study critically shows the importance of the wider implications of cardiac uptake and need of ruling out AL aetiology in each and every patient with suspect CA and evidence of cardiac uptake on bone scintigraphic imaging to avoid misdiagnosing AL with ATTR amyloidosis with fatal consequences. *Figure 3* shows a suggested diagnostic algorithm based on our findings, to be validated and implemented in larger cohorts.

The molecular basis of cardiac DPD/PYP/HMDP localization and the reason of its different behaviour between different aetiologies of CA have not yet been determined. Several studies have hypothesized that the tracer binds to amyloid deposits with a calcium-mediated mechanism.^7^ A small study performed on cardiac biopsies suggested the presence of microcalcifications in ATTR but no AL amyloidosis as a possible explanation for the exquisite sensitivity of these tracers in ATTR cardiomyopathy.^25^ However, this has not been fully assessed in larger patient populations.

Our group recently reported that ECV measured by CMR, which is an excellent non-invasive method for quantifying the cardiac extracellular space as well as a marker of disease severity in CA, shows a good correlation with the degree of cardiac ^99m^Tc-DPD uptake in ATTR amyloidosis. This suggests that the extent of amyloid infiltration, and therefore the duration of amyloid deposition, may contribute to different degrees of cardiac ^99m^Tc-DPD uptake.^20^ We found no correlation between ECV and ^99m^Tc-DPD uptake among the 61 AL patients included in the present study that underwent a gadolinium-CMR, suggesting a different mechanism in AL CA.

In our study, AL patients with cardiac ^99m^Tc-DPD uptake had higher serum cardiac biomarkers and bilirubin levels as well as poorer parameters of systolic (especially longitudinal) function than those without cardiac ^99m^Tc-DPD uptake, despite similar parameters of cardiac structure and amyloid burden, including LV wall thickness, T1, and ECV (for those who underwent CMR). This may suggest that the entity of cardiac myocyte damage rather than simple physical presence of amyloid deposits has a role and is in line with the widely accepted notion that cardiac damage in AL amyloidosis is promoted not only by the extracellular matrix expansion due to amyloid deposition, but also by a direct toxic effect exerted by light chains on cardiac myocytes, which results into greater oedema and higher T1 and T2 levels as compared to ATTR cardiomyopathy.^26^ These mechanisms cause both myocardial wall stress, resulting into a significant release of NT-proBNP, and myocytes apoptosis, resulting into a release of cardiac troponin and intracellular calcium. The released calcium accumulates within the myocardial tissue, similarly to what happens in an infarcted myocardium, which may explain why bisphosphonates can visualize both previously infarcted areas (the original purpose of PYP scintigraphy) and amyloid deposits. Since it is likely that myocardial damage (leading to local calcium accumulation) and not the amount of amyloid correlates with ^99m^DPD uptake, there is therefore a strong independent correlation with biomarkers and not DPD uptake with respect to clinical outcomes.

The above-mentioned study by Stats and Stone documented a higher concentration of macrophages in AL amyloidosis compared to the greater density of small microcalcifications within ATTR cardiac amyloid deposits, suggesting that AL patients with worse cardiac damage have greater calcium concentration within the myocardial wall and show more frequently evidence of cardiac ^99m^Tc-DPD uptake.

Among patients who underwent repeat scans, the intensity of cardiac ^99m^Tc-DPD uptake improved in 23% of cases, however, it is difficult to interpret this finding as the nature of cardiac ^99m^Tc-DPD uptake in AL amyloidosis has not been fully understood but may reflect improvement in myocardial injury.

This is the first study that investigates the prognostic role of ^99m^Tc-DPD in AL CA. As expected, most fatal events occurred within the first 6 months (109/177 total deaths). Patients with cardiac ^99m^Tc-DPD uptake tended to show worse survival compared to their counterparts without (log-rank *P* = 0.056; *Figure 2*). However, on multivariate analysis of predictors of all-cause mortality, only total bilirubin was independently associated with worse survival (*Table 3*), even when including more conventional prognostic markers in the model. Total bilirubin has been documented to be a negative prognostic in other populations with congestive heart failure. In CA, right ventricular involvement usually develops later than LV amyloid deposition and higher total bilirubin suggest a sicker population. Indeed, when right ventricular amyloid involvement occurs, prognosis worsens dramatically.^27^ An increased number of evidence suggest that impaired parameters of right ventricular function have major prognostic significance over more conventional parameters of cardiac structure and function^27^^,^^28^ and the correlation between bilirubin and all-cause mortality in our cohort confirms the importance of right ventricular dysfunction during the disease.

### Study limitations

Although this study represents the largest report of patients with AL CA ever studied with ^99m^Tc-DPD scintigraphy, the relatively small population (typical of a rare disease) might represent an unavoidable limitation.

Endomyocardial biopsy was not performed in all cases and therefore it was not possible to investigate the histological nature and difference between patients with and without cardiac ^99m^Tc-DPD uptake, including the coexistence of ATTR amyloid deposits. However, a histological confirmation of AL amyloidosis from other involved tissues (including bone marrow or abdominal fat aspiration) was reached in all cases and reported cases of coexistent AL and ATTR amyloidosis in the same patients are anecdotal.

CMR was performed in a limited number of patients, and, although no major difference between groups emerged in CMR parameters, it is not possible to generalize the results.

Only a minority of patients underwent serial ^99m^Tc-DPD scintigraphy. The present study was not designed with the purpose of understanding the role of ^99m^Tc-DPD scintigraphy to track changes over time or correlating the changes in cardiac ^99m^Tc-DPD uptake with those of other structural or functional parameters as well as haematological response to treatment. Larger prospective studies will be needed.

^99m^Tc-DPD scintigraphy at our centre is performed based on a clinical indication, especially in those cases where clinical or other instrumental findings are not *a priori* able to discriminate between AL and ATTR amyloidosis, therefore a selection bias cannot be ruled out. In particular, although our findings are consistent with previously reported cardiac ^99m^Tc-DPD uptake in AL CA, DPD findings in this cohort, with extensive cardiac involvement and poor overall survival are not necessarily representative of the general amyloidosis population presenting with more renal or liver manifestations and less extent of cardiac involvement.

Although the sensitivity and specificity of ^99m^Tc-DPD and other bone tracers are similar for diagnosing ATTR vs. AL amyloidosis, the findings in this paper cannot necessarily be extrapolated to bone imaging agents other than the ones studied (^99m^Tc-DPD).

The present analysis did not factor the impact of haematological response on the outcome, while it is recognized that very good partial response and better are associated with better outcome. However, especially among advanced stage diseased patients, which were particularly prevalent in our cohort, haematological response has not impacted outcome in significant manner.^29^

## Conclusions

^99m^Tc-DPD scintigraphy shows evidence of cardiac uptake in 40% of patients with AL CA, with at least moderate uptake in 1/4 of cases. Those with the presence of cardiac ^99m^Tc-DPD uptake tended to fare worse and had higher biomarkers consistent with worse cardiac failure/function (NT-proBNP and serum bilirubin) rather than markers of amyloid burden (ECV). A small number of patients with serial scans showed improvement in uptake which needs further investigation to understand its significance—true amyloid regression or simply resolution of ongoing myocyte damage by proteotoxic light chains.

^99m^Tc-DPD scintigraphy is widely recognized as highly accurate for identifying ATTR amyloid cardiomyopathy, in which ^99m^Tc-DPD scintigraphy usually shows a grade 2 or 3 cardiac uptake. Based on our results, in patients with suspected CA, even in presence of a grade 2 or 3 cardiac ^99m^Tc-DPD uptake, plasma cell dyscrasias should always be ruled out to avoid missed diagnoses of AL amyloidosis and potentially life-saving chemotherapy regimens.

## Supplementary data

Supplementary data are available at *European Heart Journal - Cardiovascular Imaging* online.

## SData availability

The original data are not publicly available due to need to maintain patient confidentiality.

### Funding

M.F. is supported by a BHF Intermediate Clinical Research Fellowship (FS/18/21/33447).

**Conflict of interest:** None of the authors have a confict of interest to declare for this work.

## Supplementary Material

jeab095_Supplementary_Data
