# Supplementary material for: 99mTc-DPD scintigraphy in immunoglobulin light chain (AL) cardiac amyloidosis
Source: Eur Heart J Cardiovasc Imaging. 2021 Jul 13;22(11):1304–11. doi: 10.1093/ehjci/jeab095 (PMC8527328; doi:10.1093/ehjci/jeab095)
Supplement: jeab095_Supplementary_Data [file jeab095_supplementary_data.docx]

**^99m^Tc-DPD scintigraphy in immunoglobulin light chain (AL) cardiac amyloidosis**

**Supplementary materials**

**Methods**

***Diagnostic definitions***

Diagnosis of amyloidosis was established by histological documentation of Congo red staining and demonstration of apple-green birefringence under cross-polarized light.^14^ AL amyloidosis was defined by the presence of a monoclonal plasma cell dyscrasia based on abnormal serum free light chain assay and serum/urine immunofixation, in the absence of any ATTR amyloid deposits by immunohistochemistry or mass spectrometry and in the absence of any *TTR* gene mutation (where appropriate).^15,16^ In patients with grade ≥2 ^99m^Tc-DPD cardiac uptake, when a clear-cut distinction between TTR-related and AL amyloidosis was not possible (i.e. in the absence of biopsy proven AL amyloid deposits elsewhere, including bone marrow biopsy or abdominal fat aspiration), a cardiac biopsy was performed to reach a definite etiological diagnosis.^9^

Cardiac amyloidosis was defined according to the international consensus criteria based on the presence of characteristic echocardiographic and/or CMR features suggestive of cardiac amyloidosis, as previously described.^17^ Organ involvement was otherwise based on the existing consensus criteria.^15^

***Cardiac investigations***

Twelve-lead ECGs, performed at the time of echocardiography, were reviewed for rhythm disturbances and the presence of low-voltage pattern (QRS amplitude ≤0.5 mV in all limb leads or ≤1 mV in all precordial leads).

Echocardiograms were performed using a commercially available ultrasound system (Vivid 9, GE Medical Systems, Milwaukee, WI). Analysis of the echocardiographic images was conducted offline and included both conventional (left ventricular wall thickness, ejection fraction, measures of diastolic function) as well as tissue-Doppler and speckle tracking derived measurements (mitral inflow to mitral relaxation velocity ratio (E/E’), lateral mitral systolic velocity (S’), and global longitudinal strain).^18,19^

CMRs were performed in selected cases using a standard 1.5-T clinical scanner, as previously described.^20^ All CMR images and maps were analysed offline. Features that were characteristic of amyloid were defined as the presence of diffuse subendocardial or transmural late gadolinium enhancement coupled with abnormal myocardial and blood-pool gadolinium kinetics. Native T1 mapping and post-contrast extracellular volume (ECV) were measured as described elsewhere.^20-21^

**References**

1. Gillmore JD, Maurer MS, Falk RH, Merlini G, Damy T, Dispenzieri A, Wechalekar AD, Berk JL, Quarta CC, Grogan M, Lachmann HJ, Bokhari S, Castano A, Dorbala S, Johnson GB, Glaudemans AW, Rezk T, Fontana M, Palladini G, Milani P, Guidalotti PL, Flatman K, Lane T, Vonberg FW, Whelan CJ, Moon JC, Ruberg FL, Miller EJ, Hutt DF, Hazenberg BP, Rapezzi C, Hawkins PN. Nonbiopsy Diagnosis of Cardiac Transthyretin Amyloidosis. Circulation 2016;133:2404-12.
2. Benson MD, Breall J, Cummings OW, Liepnieks JJ., Surawicz B, Knilans TK. Biochemical characterisation of amyloid by endomyocardial biopsy. Amyloid 2009;16:9-14.
3. Gertz MA, Comenzo R, Falk RH, Fermand JP, Hazenberg BP, Hawkins PN, Merlini G, Moreau P, Ronco P, Sanchorawala V, Sezer O, Solomon A, Grateau G. Definition of organ involvement and treatment response in immunoglobulin light chain amyloidosis (AL): a consensus opinion from the 10th international symposium on amyloid and amyloidosis.Am J Hematol 2005;79:319-328.
4. Lachmann HJ, Booth DR, Booth SE, Bybee A, Gilbertson JA, Gillmore JD, Pepys MB, Hawkins PN. Misdiagnosis of hereditary amyloidosis as AL (primary) amyloidosis.N Engl J Med 2002;346:1786-1791.
5. Dorbala S, Ando Y, Bokhari S, Dispenzieri A, Falk RH, Ferrari VA, Fontana M, Gheysens O, Gillmore JD, Glaudemans AWJM, Hanna MA, Hazenberg BPC, Kristen AV, Kwong RY, Maurer MS, Merlini G, Miller EJ, Moon JC, Murthy VL, Quarta CC, Rapezzi C, Ruberg FL, Shah SJ, Slart RHJA, Verberne HJ, Bourque JM. ASNC/AHA/ASE/EANM/HFSA/ISA/SCMR/SNMMI Expert Consensus Recommendations for Multimodality Imaging in Cardiac Amyloidosis: Part 2 of 2-Diagnostic Criteria and Appropriate Utilization. J Card Fail. 2019:25:854-865.
6. Lang RM, Bierig M, Devereux RB, Flachskampf FA, Foster E, Pellikka PA, Picard MH, Roman MJ, Seward J, Shanewise JS, Solomon SD, Spencer KT, Sutton MS, Stewart WJ; Chamber Quantification Writing Group; American Society of Echocardiography's Guidelines and Standards Committee; European Association of Echocardiography. Recommendations for chamber quantification: a report from the American Society of Echocardiography's Guidelines and Standards Committee and the Chamber Quantification Writing Group, developed in conjunction with the European Association of Echocardiography, a branch of the European Society of Cardiology.J Am Soc Echocardiogr 2005;18:1440-1463.
7. Quarta CC, Solomon SD, Uraizee I, Kruger J, Longhi S, Ferlito M, Gagliardi C, Milandri A, Rapezzi C, Falk RH. Left ventricular structure and function in transthyretin-related versus light-chain cardiac amyloidosis. Circulation 2014;129:1840-9.
8. Martinez-Naharro A, Treibel TA, Abdel-Gadir A, Bulluck H, Zumbo G, Knight DS, Kotecha T, Francis R, Hutt DF, Rezk T, Rosmini S, Quarta CC, Whelan CJ, Kellman P, Gillmore JD, Moon JC, Hawkins PN, Fontana M. Magnetic Resonance in Transthyretin Cardiac Amyloidosis. J Am Coll Cardiol 2017;70:466-477.
9. Martinez-Naharro A, Kotecha T, Norrington K, Boldrini M, Rezk T, Quarta C, Treibel TA, Whelan CJ, Knight DS, Kellman P, Ruberg FL, Gillmore JD, Moon JC, Hawkins PN, Fontana M. Native T1 and Extracellular Volume in Transthyretin Amyloidosis. JACC Cardiovasc Imaging 2019;12:810-819.
